# Current status of research on the risk factors and pathogenesis of metabolic dysfunction-associated steatotic liver disease

**DOI:** 10.3389/fendo.2026.1819756

**Published:** 2026-04-16

**Authors:** Dan Chen, Lingling Yan, Tong Wu, Junpeng Zhao, Xuebing Xu, Weisong Xu, Hong Zhang, Mingbing Xiao

**Affiliations:** 1 Department of Gastroenterology, Affiliated Hospital of Nantong University, Medical School of Nantong University, Nantong, Jiangsu, China; 2 Department of Internal Medicine, Qidong Second People’s Hospital, Nantong, Jiangsu, China; 3 Department of Gastroenterology, Affiliated Nantong Rehabilitation Hospital of Nantong University, Nantong, Jiangsu, China; 4 Department of Critical Care Medicine, Haimen District People’s Hospital, Nantong, Jiangsu, China; 5 Research Center of Clinical Medicine, Affiliated Hospital of Nantong University, Nantong, Jiangsu, China

**Keywords:** MASLD, metabolic dysfunction, pathogenesis, risk factors, T2DM

## Abstract

Metabolic Dysfunction-Associated Steatotic Liver Disease (MASLD) is a chronic metabolic liver disorder characterized by excessive hepatic fat accumulation in humans and is closely associated with metabolic dysfunction. MASLD has become a global public health issue that cannot be ignored. Currently, the global prevalence of MASLD has reached as high as 38%. It can evolve from simple fatty liver disease through liver fibrosis and cirrhosis, ultimately potentially leading to hepatocellular carcinoma. In 2019, approximately 134,000 people worldwide died from complications related to this disease. In China, the impact of this disease is even more pronounced, having surpassed that of viral hepatitis to become the leading cause of cirrhosis. Faced with this serious public health challenge, this review systematically organizes the core risk factors and major pathogenesis mechanisms of MASLD. This study aims to provide critical scientific evidence for advancing liver disease prevention and management strategies, reducing the disease burden, and realizing precise diagnosis and stratified treatment. Unlike conventional reviews that discuss risk factors and pathogenic mechanisms separately, this review integrates these elements into a unified pathophysiological framework, providing a more comprehensive understanding of MASLD.

## 1 Introduction

Metabolic dysfunction-associated steatotic liver disease (MASLD) is a chronic condition affecting the liver. This disease was previously referred to as nonalcoholic fatty liver disease (NAFLD) in past guidelines. It has a close connection with metabolic abnormalities. The “NAFLD” nomenclature has been a subject of ongoing debate among the international hepatology community for years. A primary point of criticism is its overly exclusive definition. Another key concern is that it fails to capture the core driving factors of the disease (1). The international community sought to address the flaws in the “NAFLD” nomenclature. To this end, a Delphi consensus was formally published (2). To refine the disease terminology system, this consensus was jointly spearheaded by hepatology societies, endocrinology associations, and patient advocacy organizations from 56 countries. The majority of experts in the consensus development panel firmly advocated for the adoption of specialized terminology that accurately reflects the pathophysiological mechanisms of the disease. Following careful review, the panel did not select these options. Instead, they finalized MASLD as the official replacement for NAFLD. The new definition clearly delineates MASLD as a condition characterized by hepatic steatosis. One of the key diagnostic criteria for MASLD is that patients must have at least one cardiometabolic risk factor (CMRF). Importantly, the diagnosis of this disease requires the exclusion of all other identifiable steatosis-causing factors (3). This terminological shift has led to two critical advances. First, it more accurately captures the inherent connection of the disease to systemic metabolic dysfunction. Second, it creates a foundation for developing precision diagnosis and management strategies rooted in causal mechanisms. Collectively, these changes mark a pivotal turning point in how we understand this liver condition.

MASLD disproportionately affects three key groups: males, obese individuals, and those living with type 2 diabetes (T2DM). Globally, this condition affects approximately 38% of the adult population (4). MASLD has become a serious public health challenge because of its diverse clinical manifestations and progressive nature. The clinical spectrum of MASLD is broad. Without intervention, the disease’s continuous progression may lead to severe hepatic outcomes, such as liver fibrosis, cirrhosis, and even the development of hepatocellular carcinoma. Statistical data show that approximately 134,000 people worldwide died from MASLD-related complications in 2019 (5). Cirrhosis and liver cancer were the main causes of these deaths. In addition, the overall impact of MASLD on public health continues to expand. MASLD is currently the most prevalent cause of chronic liver disease (6). Furthermore, this condition significantly elevates patients’ subsequent risk of developing cirrhosis and hepatocellular carcinoma (HCC) (7). China has one of the highest cirrhosis disease burdens in the world. For this reason, MASLD needs special attention in the country. From 1990--2021, the number of cirrhosis cases in China significantly increased, with the total number of patients increasing from approximately 9.67 million to nearly 10.99 million. In 2022, the number of new cases of HCC reached 367,700 (standardized incidence rate: 15.2 per 100,000 people) (8). As lifestyles change, the influence of metabolic risk factors on liver disease becomes stronger than ever before. MASLD has now surpassed viral hepatitis as the leading cause of liver cirrhosis in China. Research by Li et al. revealed a significant increase in the proportion of patients who progressed from MASLD to cirrhosis. In 1990, MASLD-related cirrhosis accounted for 44.3% of all cirrhosis cases. By 2019, this proportion had increased to 68.6% (9). At the same time, the study also offers another key finding. In recent years, the disease burden associated with liver cirrhosis in China has shown a downwards trend, with both age-standardized mortality rates and disability-adjusted life years decreasing. This may reflect overall improvements in clinical diagnosis and treatment. However, the real impact of the disease could be worse than current numbers suggest. Patients with compensated cirrhosis do not usually receive a liver biopsy.

To address this severe public health challenge, translating scientific knowledge into effective prevention and control strategies has become an urgent priority. Therefore, this review systematically summarizes the core risk factors for MASLD (including obesity, features of non-obese populations, type 2 diabetes, and genetic susceptibility) and analyzes its major pathogenic pathways in depth, focusing on three key areas: imbalance in hepatic lipid metabolism homeostasis, immune inflammation activation, and disruption of the gut–liver axis. This summary can help China improve its liver disease prevention and control plans. It can also reduce the overall disease burden. Hence, this work holds significant practical and clinical value. Unlike previous reviews that primarily describe risk factors and pathogenic mechanisms independently, this review aims to integrate these aspects into a unified framework. By linking metabolic risk factors with key biological pathways, we propose a continuous pathophysiological model of MASLD. In addition, this review highlights emerging perspectives, including lean MASLD and population-specific genetic susceptibility, particularly in Asian populations, to provide a more comprehensive and clinically relevant understanding of disease heterogeneity.

## 2 Research progress on risk factors for MASLD

**Figure 1:**
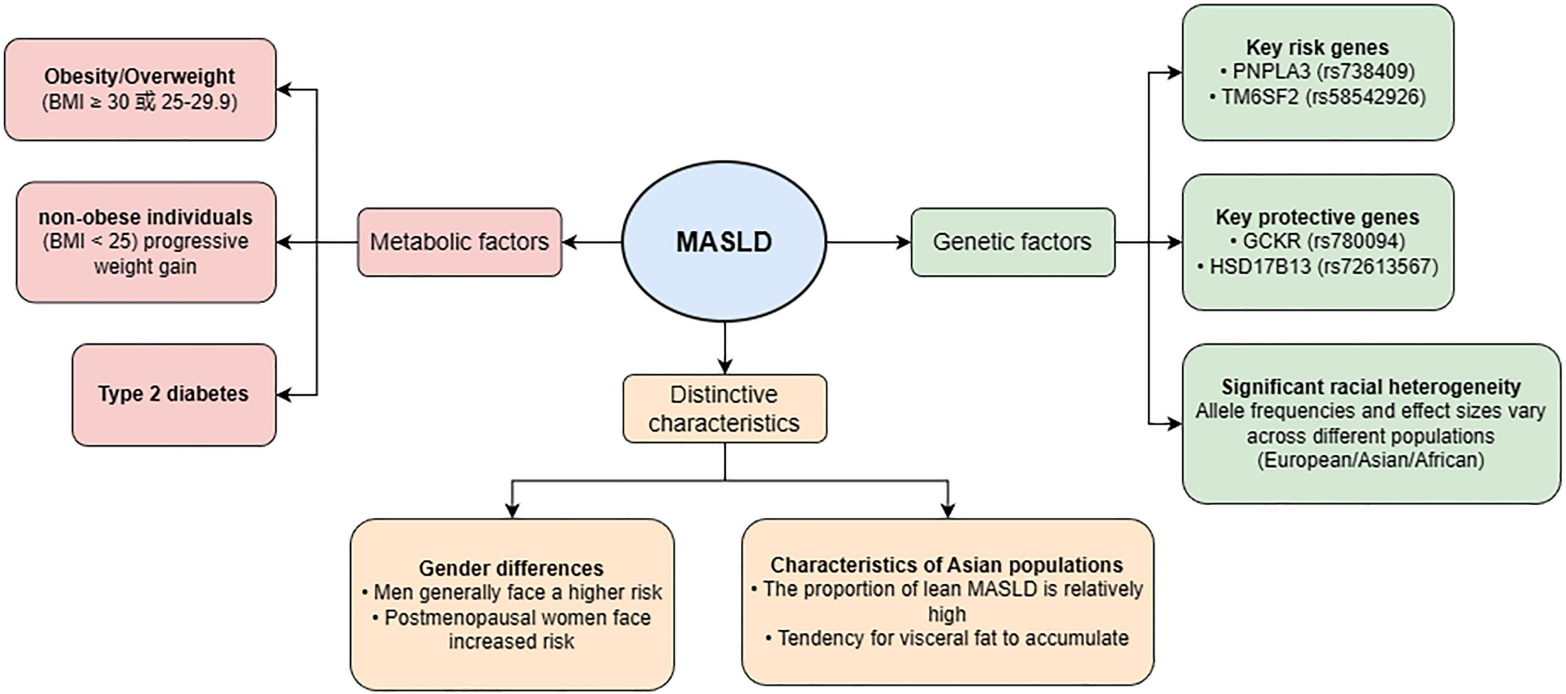
Risk factors for MASLD.

Risk factors for MASLD are not independent from its underlying pathogenic mechanisms but represent upstream drivers of disease development. Many metabolic abnormalities—particularly insulin resistance, dyslipidemia, and disturbances in lipid metabolism—act both as clinical risk factors and as key mediators initiating the core pathogenic processes discussed later, including lipid accumulation, inflammation, and fibrosis. Therefore, the following sections describe these factors as part of a continuous pathophysiological spectrum rather than isolated entities (**Figure 1**).

### 2.1 Obesity individuals

The definition of obesity was a body mass index (BMI) ≥ 30. Obesity is classified as a chronic disease by the World Health Organization (WHO) (10). This is caused by the complex ways in which genetics, neurobiology, lifestyle, and environmental factors work together. Over the past few decades, with rapid economic and social development, changes in dietary patterns, and a general decline in physical activity levels, obesity has continued to worsen worldwide. The global obese population has now surpassed one billion, with obesity rates rising in nearly every country (11). According to a 2023 report by the World Obesity Federation, if no effective interventions are implemented, the global obese population is projected to reach approximately 2 billion by 2035. The report further predicts that childhood and adolescent obesity rates will surge significantly, with the number of obese adolescent males projected to increase by 100% to 208 million, whereas the number of obese adolescent females may rise by 125% to 175 million (12, 13). Currently, obesity is widely recognized as one of the major causative factors of MASLD (14). An increasing number of people are currently dealing with overweight or obesity. Jamialahmadi and colleagues performed a clinical analysis using data from the UK Biobank. The results indicate that visceral adipose tissue (VAT), whole-body fat mass (WFM), and body mass index (BMI) can all serve as independent predictors for assessing the risk of developing three types of liver health issues. These issues include hepatic triglyceride deposition (PDFF), inflammation, and fibrosis (cT1). Among these three factors, VAT has the strongest connection with liver problems (15). One key finding concerning obesity and liver health stands out. Obesity increases the genetic tendency to cause steatotic liver disease (SLD). It also worsens hepatic steatosis. The mechanism by which obesity causes these issues involves disrupting lipid balance and creating metabolic problems. It also triggers the systemic subtype of MASLD. This subtype is closely associated with an increased risk of systemic metabolic complications. Furthermore, the low-grade chronic inflammation associated with obesity can directly promote hepatic fat accumulation and the progression of fibrosis. It can also induce metabolic disturbances, such as insulin resistance, dyslipidemia, and hyperglycemia. The aforementioned pathological mechanisms collectively constitute the key elements in the development and progression of MASLD. Recent studies have shown that the prevalence of MASLD is significantly greater among overweight and obese individuals than in the general population. Additionally, a strong correlation exists between obesity severity and two liver-related factors. These factors are hepatic fat content (assessed by CAP values) and liver stiffness (16).

A recent meta-analysis examined a cohort of 101,028 overweight or obese individuals. This study further quantified how obesity harms liver health, showing that it has a clear negative effect on the development of MASLD (17). In overweight individuals, the prevalence of MASLD is approximately 69.99%, and it further increases to 75.27% in obese individuals. Additionally, MASH affects approximately 33.5% to 33.7% of the people in both groups. Notably, in this patient group, approximately 20% of those with MASLD who were overweight or obese had clinically significant liver fibrosis (stages F2--4). Among them, close to 7% had progressed to advanced fibrosis (stages F3--4). Research by Jaideep et al. also confirmed that BMI is a key factor in predicting outcomes in MASLD (18). The study revealed a positive dose–response relationship between BMI and MASLD incidence. This means that the greater a person’s BMI is, the greater the likelihood of developing MASLD. The highest risk appears in patients with severe obesity, whose BMI is 50 kg/m^2^ or higher. Compared with overweight individuals (BMI 25 to less than 30 kg/m^2^), severely obese patients face several elevated risks. These patients have a 2.27-fold greater risk of hepatic decompensation, a 1.29-fold greater risk of obesity-related extrahepatic cancer, and a 3.24-fold greater risk of all-cause mortality. All these risks are significantly greater than those reported in lean MASLD patients, who have a BMI below 25 kg/m^2^. Additionally, higher BMI was associated with a J-shaped correlation with the risk of liver decompensation and obesity-related cancers. These associations remained strong even after confounders, including age and sex, were adjusted for. These findings confirm that obesity plays two major roles in MASLD. It is a key predisposing factor for this disease. It is also a core driver that leads to independent disease progression and negative health outcomes.

### 2.2 Nonobese individuals

Notably, there are also patients with “lean MASLD” (19). Studies have revealed that the risk factors for nonobese MASLD patients (BMI < 25 kg/m^2^) are multifactorial. The core factors include adult weight change, sex differences, metabolic abnormality-related indicators, lifestyle factors, and genetic and physiological factors (20). Metabolic dysfunction is central to the diagnosis of MASLD. Even without obesity, having related metabolic issues alone greatly increases the risk of developing the disease. Fukamizo et al. identified the core risk factor for MASLD in nonobese people as weight change in adulthood (21). Weight gain after the age of 20 is particularly impactful. The risk of developing the disease increases in a dose-dependent manner with this weight gain. In addition, dyslipidemia is more common among lean MASLD patients and is characterized by elevated triglycerides and lower levels of low-density lipoprotein cholesterol. These higher levels make their disease risk even worse. Individuals with weight gain of 10 kilograms or more exhibit a significantly increased incidence of MASLD. The average increase in risk was 6.6%, and this increase varied from 2% to 19% across different people. In contrast, maintaining stable weight (within a 3-kilogram range) or losing weight (more than 3 kilograms) was associated with a reduced incidence of the disease. Among specific groups, individuals who are not obese face a higher incidence of MASLD. Male individuals fall into this high-risk category, with an RD of 10.5%. This is notably higher than the 4.5% RD observed in females. Additionally, nonobese individuals who have certain comorbidities are more susceptible. These comorbidities include abdominal obesity, dyslipidemia, hyperuricemia, and elevated ALT levels. When these risk factors occur together, the associated risk difference for MASLD linked to weight gain could reach 18.1%. Weight gain can cause metabolic abnormalities. These problems include insulin resistance and increased free fatty acids. These abnormalities alone increase the risk of MASLD in nonobese people. Two more factors increase the degree of risk. Asian populations have a unique predisposition to visceral fat accumulation. Genetic polymorphisms such as PNPLA3 also contribute to increased risk. A Japanese longitudinal observational study focused on nonobese individuals. Moreover, a high BMI is one of the key factors contributing to the onset of MASLD (22). For both men and women, there is a clear connection. Increasing BMI over time is significantly associated with an increased risk of MASLD. The analysis was adjusted for baseline age, lifestyle habits, and other factors. After these adjustments, the OR was 1.90 (95% CI: 1.64--2.19) for men and 1.95 (95% CI: 1.72--2.21) for women. This association has been shown to be independent. It is not influenced by other cardiometabolic factors. This is an important observation. Furthermore, a rise in BMI was linked to unfavorable changes in several health indicators. These included a larger waist size, higher triglyceride levels, and lower HDL cholesterol. This pattern further suggests that increasing BMI is a major factor contributing to the development and progression of MASLD, even in nonobese individuals. Therefore, weight loss and dietary changes are important components of MASLD treatment. This applies to all patients, including those who are obese and those who are lean.

### 2.3 T2DM

It is projected that by 2035, nearly 600 million people worldwide will be affected by type 2 diabetes, but there are significant variations in the prevalence rates across different regions (23). In 2021, China’s age-standardized prevalence rate of T2DM among adults was approximately 9.96%, which was 2.5 times greater than that in 1990. It is projected to increase to 18.17% by 2050 (24), indicating that the disease burden continues to intensify. There appears to be a bidirectional association between MASLD and T2DM: an increase in the prevalence of T2DM is associated with an increase in the occurrence of MASLD, and vice versa. According to the results of a genome-wide association study (GWAS), MASLD and T2DM share significant genetic overlap, with 115 common susceptibility genes identified between the two conditions. By analyzing differential gene expression, 15 core genes associated with both T2DM and MASLD were identified. Additionally, a significant positive causal association between MASLD and T2DM was revealed via bidirectional Mendelian randomization (MR) analysis (25). On the basis of existing epidemiological trends and shared genetic underpinnings, the close clinical association between T2DM and MASLD has been confirmed by numerous studies. Synthesizing findings from multiple clinical investigations, T2DM is undoubtedly a core driver of the development and progression of MASLD. First and foremost, epidemiological data provide the most direct evidence. According to a meta-analysis incorporating 156 studies and covering nearly 1.8 million patients with type 2 diabetes, the global prevalence of MASLD in this population is approximately 65% (95% CI: 62%-68%), whereas the prevalence of MASH is approximately 32% (95% CI: 17%-51%) (26). Research from studies in China also indicates that the prevalence of MASLD in the T2DM population is as high as 61.3%, which is significantly higher than that in the non-T2DM population (27). After accounting for factors such as age and obesity, T2DM continues to be an independent risk factor for MASLD (28). In patients with T2DM who also have hyperferritinemia, the rate of MASLD increases even more, reaching 78.5%. The risk of developing severe liver scarring is also significantly greater in these patients, at 35.5%, than in those without this condition, at 22.1% (29). These patients face a greater risk of adverse health outcomes. Two common negative results they are more likely to experience are elevated liver enzymes and the progression of liver fibrosis.

### 2.4 Genetic susceptibility

Large-scale genome-wide association studies (GWASs) in recent years have successfully identified numerous genetic variants associated with increased hepatic fat content and increased liver volume (30). This finding further confirms that, in addition to clinical factors, genetic factors play a significant role in determining an individual’s susceptibility to developing fatty liver disease. A key point is the substantial variation in these genetic factors across different ethnic groups. Risk or protective alleles are not distributed equally among global populations. For example, a variant in the HSD17B13 gene (rs72613567) is a well-known protective factor against liver fibrosis in people of European descent (31). However, its benefits against liver inflammation and scarring might only apply to specific MASLD subgroups, such as patients with more severe obesity or diabetes (32). In Chinese MASLD patients, this variant was found in 34% of individuals, but its effects on metabolism can vary significantly (33). Analysis of multiple genetic risk loci revealed that people of African ancestry tend to carry a lower overall genetic risk for MASLD. In contrast, Hispanic populations often have the highest genetic risk burden, whereas those of European ancestry have a moderately elevated risk (34). These findings indicate that genetic factors strongly influence how MASLD is distributed worldwide. These genetic differences underscore the urgent need for more research focused on specific ethnic and regional groups. These studies have been crucial for understanding the unique pathogenesis of MASLD in Asian populations.

Within this context, GWASs have pinpointed several key risk genes that are significant for Asian populations. Wang et al. reported that certain alleles of the PNPLA3 gene (such as rs738409) and the SAMM50 gene (such as rs2235776) are important risk factors in East Asian groups, including those in Taiwan, China. Notably, the PNPLA3 variant is considered a key genetic factor for MASLD even in individuals who are not obese (35). Separately, research by Kwon et al. revealed that specific variants in the GCKR gene (rs780094 and rs1260326) have a protective effect in Korean and Han Chinese populations. These variants can lower the risk of developing the disease by 12% to 25% (36). Furthermore, a study by Dai et al. revealed that protective genotypes of the HSD17B13 (rs72613567) and KLB (rs12152703) genes can significantly reduce the risk of MASLD and related conditions, such as carotid artery plaques, with an odds ratio of 0.33. In contrast, risk genotypes such as one in the TM6SF2 gene (rs58542926) substantially increase disease risk (37). These genetic variants do not act alone. They influence disease risk independently and interact with environmental factors such as diet. Together, they shape a person’s individual susceptibility to MASLD and its different clinical forms. Therefore, the genetic background is crucial for understanding how MASLD develops. This information helps explain why the disease varies so much between individuals and paves the way for more targeted prevention strategies. To facilitate comparison across major risk profiles, a summary of key characteristics is presented in **Table 1**.

**Table 1:** Comparison of major risk profiles in MASLD.

Characteristics	Obese MASLD	Non-obese (lean) MASLD	T2DM-associated MASLD	Genetic susceptibility
BMI	≥30 kg/m^2^	<25 kg/m^2^	Variable	Variable
Core feature	Excess adiposity	Metabolic dysfunction without obesity	Hyperglycemia & insulin resistance	Genetic polymorphisms
Key metabolic abnormalities	Insulin resistance, dyslipidemia, inflammation	Dyslipidemia, visceral fat accumulation	Severe insulin resistance, glucose dysregulation	Lipid metabolism alterations
Main drivers	Adipose tissue expansion, chronic inflammation	Weight gain, visceral adiposity, metabolic imbalance	Hyperinsulinemia, glucotoxicity, lipotoxicity	PNPLA3, TM6SF2, GCKR, etc.
Pathogenic links	Lipid accumulation, inflammation, fibrosis	Similar mechanisms but less BMI-dependent	Accelerated fibrosis and disease progression	Modulates susceptibility and severity
Clinical characteristics	High prevalence, strong BMI correlation	Often underdiagnosed	High prevalence (~60–70%)	Ethnic variability
Disease progression risk	Moderate to high	Variable but significant	High (fibrosis, MASH)	Influences progression risk
Special notes	Strongly associated with metabolic syndrome	Common in Asian populations	Bidirectional relationship with MASLD	Population-specific differences

## 3 Research progress on the pathogenesis of MASLD

The pathogenic mechanisms of MASLD arise directly from the metabolic disturbances described above. Conditions such as obesity, type 2 diabetes, and metabolic dysfunction initiate and sustain a series of interconnected biological processes, including lipid metabolism imbalance, immune-inflammatory activation, and disruption of the gut–liver axis. These mechanisms therefore represent the downstream biological consequences of upstream metabolic risk factors, forming a continuous and integrated pathophysiological continuum.

### 3.1 Imbalance in hepatic lipid metabolism homeostasis

**Figure 2:**
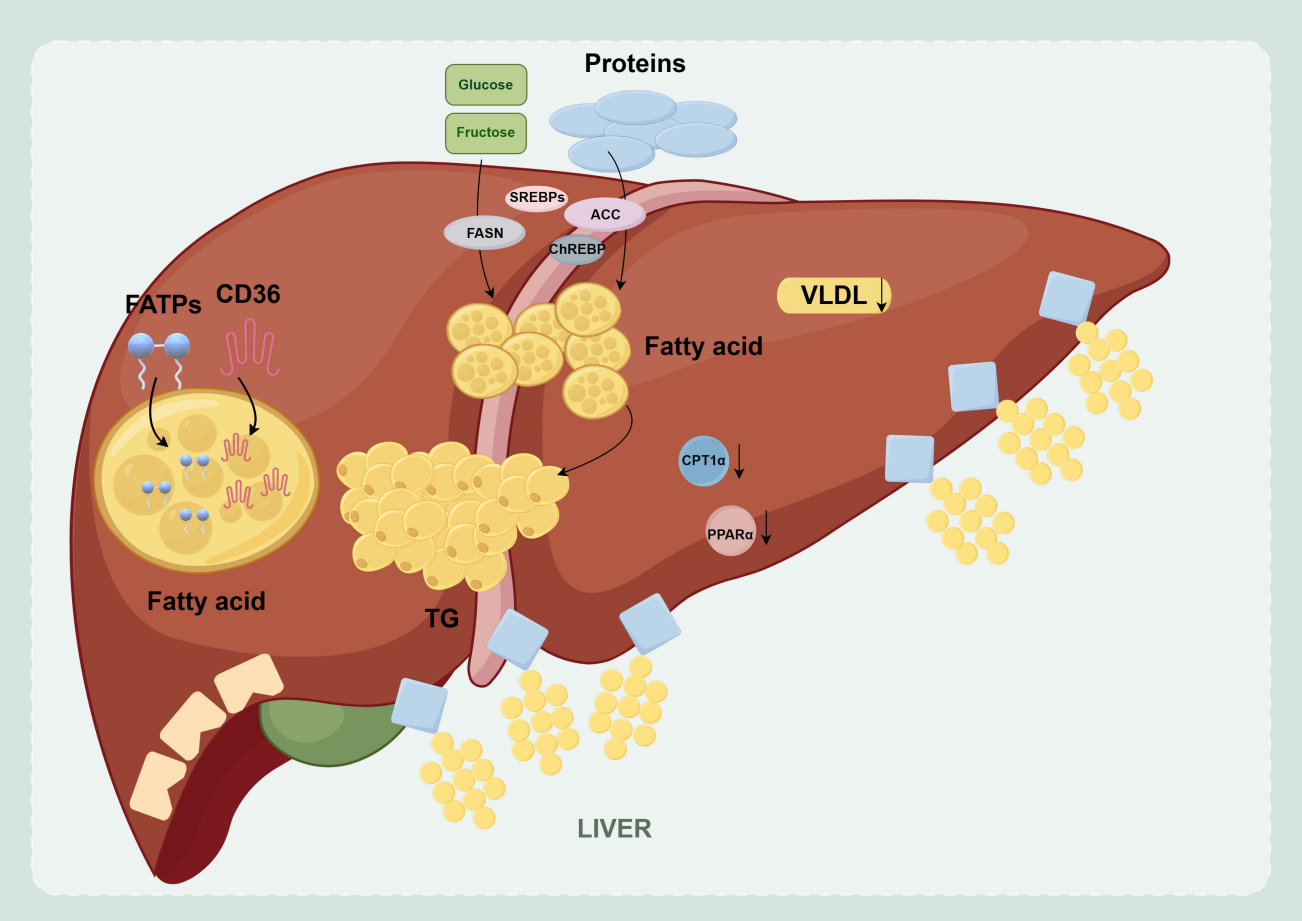
Imbalances in hepatic lipid metabolism homeostasis.

Pathologically, MASLD is characterized by the abnormal accumulation of numerous lipids in the hepatocyte cytoplasm. These cellular structures store harmless fats as well as damaging types of fat. The damage types can turn on stress pathways inside the cell. Therefore, at its core, MASLD is a disease in which the liver loses its ability to properly regulate and balance its fat levels (38). This metabolic disturbance is caused mainly by dynamic imbalances in several key lipid metabolic pathways (39) (**Figure 2**). First, there is a significant enhancement in fatty acid uptake and transport, which is mediated by transporters such as coreceptor 36 (CD36) and fatty acid transport proteins (FATPs). Second, there is an upregulation of the *de novo* lipogenesis (DNL) pathway, which is regulated by transcription factors, including sterol regulatory element-binding proteins (SREBPs) and carbohydrate response element-binding proteins (ChREBPs), as well as key enzymes such as acetyl-CoA carboxylase (ACC) and fatty acid synthase (FASN). Third, impaired fatty acid β-oxidation is correlated primarily with the downregulation of peroxisome proliferator-activated receptor α (PPARα) and the reduced activity of carnitine palmitoyltransferase 1α (CPT1α). Fourth, lipid transport is reduced, as shown by insufficient very low-density lipoprotein (VLDL) secretion. All these imbalances cause too many lipids to accumulate in hepatocytes. This buildup then triggers lipotoxicity, inflammation, and fibrosis. These alterations are largely driven by upstream metabolic disturbances, including insulin resistance and dyslipidemia, as discussed in the context of risk factors. These metabolic disturbances also contribute to mitochondrial dysfunction and oxidative stress, further aggravating hepatocellular injury.

#### 3.1.1 Regulation of fatty acid oxidation

Impaired fatty acid β-oxidation represents a critical mechanism underlying hepatic lipid accumulation in MASLD. Under normal conditions, fatty acid oxidation serves as a major pathway for lipid clearance and energy production in hepatocytes. In MASLD, this process is significantly reduced, leading to lipid accumulation and metabolic imbalance. This impairment is partly mediated by decreased activity of regulatory factors such as peroxisome proliferator-activated receptor α (PPARα), which plays a central role in promoting fatty acid oxidation. Reduced PPARα signaling diminishes the expression of key enzymes involved in mitochondrial β-oxidation, including carnitine palmitoyltransferase 1α (CPT1α), thereby limiting lipid utilization. At the same time, suppression of fatty acid oxidation is often accompanied by enhanced lipogenesis, further aggravating lipid accumulation. Overall, disruption of fatty acid oxidation reflects a fundamental imbalance between lipid synthesis and degradation and represents a key pathogenic process in MASLD, rather than the effect of a single molecular regulator (40–44).

#### 3.1.2 Regulation of *de novo* lipogenesis

Enhanced *de novo* lipogenesis is another major contributor to hepatic lipid accumulation in MASLD. Under conditions of metabolic dysfunction, particularly insulin resistance and overnutrition, hepatic lipid synthesis is markedly increased, leading to excessive production of triglycerides and cholesterol. This process is regulated by transcription factors such as sterol regulatory element-binding proteins (SREBPs), which are activated through signaling pathways including mTORC1. Activation of these pathways promotes the expression of key lipogenic enzymes, such as acetyl-CoA carboxylase (ACC) and fatty acid synthase (FASN), thereby increasing lipid synthesis. Meanwhile, impaired lipid export and reduced fatty acid oxidation further exacerbate lipid accumulation within hepatocytes. The resulting lipid overload generates a toxic intracellular environment, inducing oxidative stress and inflammatory responses through the production of reactive oxygen species (ROS) and proinflammatory cytokines such as TNF-α and IL-6. These processes promote hepatocyte injury and activate hepatic stellate cells, thereby contributing to fibrosis progression. Taken together, dysregulated lipogenesis represents a central mechanism linking metabolic risk factors with hepatic steatosis, inflammation, and fibrosis, highlighting its critical role in MASLD pathogenesis (45–47).

### 3.2 Immune inflammation activation

Immune and inflammatory activation is a central mechanism that causes MASLD to worsen, progressing from simple fat accumulation to MASH and liver fibrosis. This process often starts when free fatty acids and other harmful metabolic substances activate a receptor called Toll-like receptor 4 (TLR4) on liver cells, including hepatocytes and Kupffer cells. This activation, which relies on a key signaling protein called MyD88, sets off a chain of downstream signals. It prompts the release of proinflammatory signaling proteins, such as TNF-α and IL-6 (48, 49). Other inflammatory proteins, such as IL-17A and IL-17F, work together with TLR4 to further intensify this inflammatory response. This not only exacerbates fat build-up in liver cells by increasing fat production and slowing fat breakdown but also directly activates HSCs, which drive the formation of scar tissue. Furthermore, body-wide markers of immune inflammation show important links to the severity of the disease. Systemic inflammatory markers, such as the platelet-to-lymphocyte ratio and systemic immune-inflammatory indices, have significant evaluative value. The levels of these genes are strongly correlated with both the severity of hepatic steatosis and the risk of developing liver fibrosis (50, 51). In patients with severe steatosis, PIV is positively correlated with the SII and the C-reactive protein-to-albumin ratio (CAR). Moreover, in those with mild to moderate steatosis, the SII is closely related to the level of IL-17A. When this inflammatory state continues over time, it ultimately leads to damage to liver cells, excessive fat accumulation, and progressive scarring. This sequence of events drives the disease forward from simple hepatic steatosis to more severe stages of MASLD (52).

Moreover, Supruniuk et al. explicitly demonstrated that the core driving mechanism underlying the onset and progression of MASLD lies in the imbalance between the helper T (Th17) and regulatory T (Treg) cells, along with the dysregulation of related cytokines (53). In MASLD patients, particularly those with advanced disease, the proportion of CD4+IL-17+ Th17 cells in the peripheral blood is significantly elevated, reaching levels up to twice those observed in healthy individuals. In contrast, the number of CD4+CD25+Foxp3+ Tregs was reduced. This disrupts the proinflammatory and anti-inflammatory immune balance. This imbalance becomes more severe as the disease progresses. It worsens when progressing from early simple steatosis to advanced inflammatory steatosis. The levels of proinflammatory cytokines secreted by Th17 cells are elevated. These include IL-17A and IL-22. IL-17A is positively correlated with aspartate aminotransferase (AST), a marker of liver injury. IL-22 is associated with BMI, the obesity index. Together, these two cytokines promote hepatocellular inflammation, lipid accumulation, and HSC activation. On the other hand, there is a significant reduction in the expression level of IL-10, which is secreted by regulatory T cells. This change directly leads to a decrease in the IL-10/IL-17A and IL-10/IL-22 ratios, thereby significantly weakening the body’s anti-inflammatory effects. Moreover, this immunoinflammatory state is linked to two metabolic issues. One is insulin resistance. The other is elevated levels of LDL-C. These metabolic problems further worsen hepatic metabolic disorders. Eventually, this entire process pushes MASLD to progress to MASH and hepatic fibrosis. Therefore, immunoinflammatory activation acts as a key pathological link. It connects metabolic disorders to progressive hepatic injury.

### 3.3 Disruption of the gut–liver axis 

The gut–liver axis refers to the close interaction between the liver and the intestines. This system controls the flow of nutrients, microbial antigens, metabolites, and bile acids. It also shapes metabolic and immune responses in both organs. In addition, the gut–liver axis influences the composition and functionality of microbial communities in the gut and liver, which in turn interact with each other.

In MASLD, the role of bile acids (BAs) extends well beyond their basic function in digestion. Keeping their transport and synthesis tightly controlled is very important. When this control fails, BAs build up, which is connected to the toxic and inflammation-promoting effects observed in MASLD (54). This dysregulation occurs when key synthetases (such as Cyp7a1), regulatory receptors (such as FXR), and transporters (such as NTCP and BSEP) do not work properly. This disrupts how BAs are made, recycled between the liver and intestines, and secreted. As a result, the levels of total bile acids and important subtypes (such as UDCA, TCA, and CDCA) rise in the blood. When BAs accumulate excessively within the liver, they further trigger activation of the TLR4–NF-κB pathway. This process promotes the massive release of proinflammatory cytokines such as TNF-α and IL-6, significantly exacerbating hepatocyte damage. Concurrently, these released proinflammatory factors directly activate hepatic stellate cells, driving their phenotypic transformation into myofibroblasts. This promotes the buildup of scar tissue. Notably, the levels of conjugated bile acids such as TCA and TLCA are significantly greater in patients with MASH, which strengthens this damaging process. Furthermore, the disrupted interaction between BAs and gut bacteria exacerbates the metabolic imbalance. This creates a damaging cycle in which metabolic problems, inflammation, and fibrosis fuel each other, strongly affecting how MASLD progresses (55).

**Figure 3:**
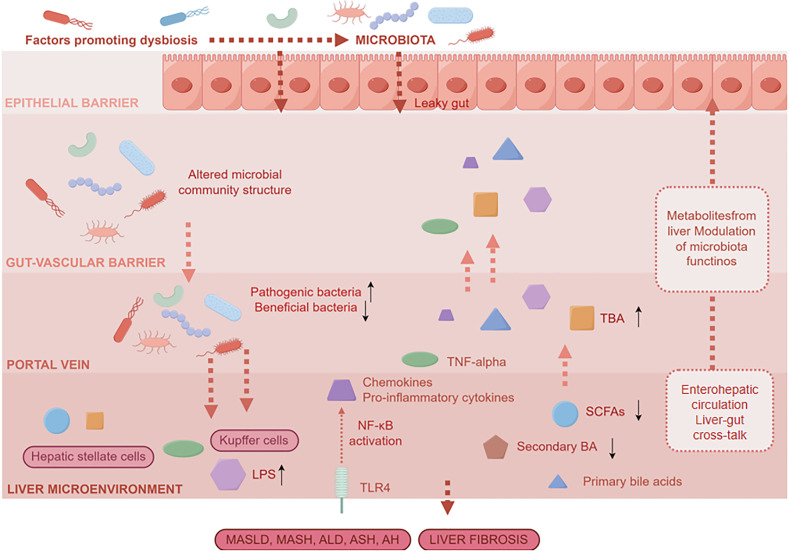
Dysbiosis of the gut microbiota interacts with bile acid metabolism disorders to cause liver damage.

Moreover, the intricate gut microbiota is vital for nutrient absorption and establishing a healthy immune system (56). Patients with MASLD often have an imbalance in their gut microbiota. This imbalance changes how bile acids are processed, affecting their signaling along the gut–liver axis. It blocks the activation of a key receptor called FXR, which worsens fat buildup in the liver. This imbalance also suppresses anti-inflammatory signaling pathways and compromises intestinal barrier integrity, leading to increased intestinal permeability. As a result, bacteria and their metabolic byproduct LPS can migrate through the portal vein to the liver. LPS subsequently triggers the TLR4–NF-κB signaling pathway in the liver, inducing the release of proinflammatory cytokines such as TNF-α and IL-6. These changes collectively exacerbate hepatocyte injury and activate hepatic stellate cells, thereby driving the progressive development of hepatocellular carcinoma (57). This imbalance in the gut microbiota promotes MASLD through two main connected mechanisms (58). First, the shift toward more proinflammatory and fewer beneficial bacteria weakens the intestinal wall. This increased permeability allows LPS and other toxins into the bloodstream, where they trigger widespread inflammation via TLR4 and fuel liver inflammation and fat accumulation through proteins such as IL-17 and TNF-α. Second, the imbalance disrupts the normal metabolism of nutrients. It reduces the production of helpful metabolites, such as short-chain fatty acids (SCFAs). Lower SCFAs weaken the protective lining of the gut and affect genetic regulation processes, further disturbing the balance of energy and fat metabolism in the liver. In the end, the combined effect of this immune dysfunction and disrupted nutrient balance promotes fat accumulation, inflammation, and scarring in the liver, driving both the onset and worsening of MASLD (**Figure 3**).

### 3.4 Mitochondrial dysfunction, oxidative stress, and endoplasmic reticulum stress

In addition to the mechanisms described above, mitochondrial dysfunction, oxidative stress, and endoplasmic reticulum (ER) stress are recognized as key contributors to the progression of MASLD (59). These processes are closely interconnected with lipid metabolism imbalance and immune-inflammatory activation, forming an integrated pathogenic network rather than independent pathways.

**Figure 4:**
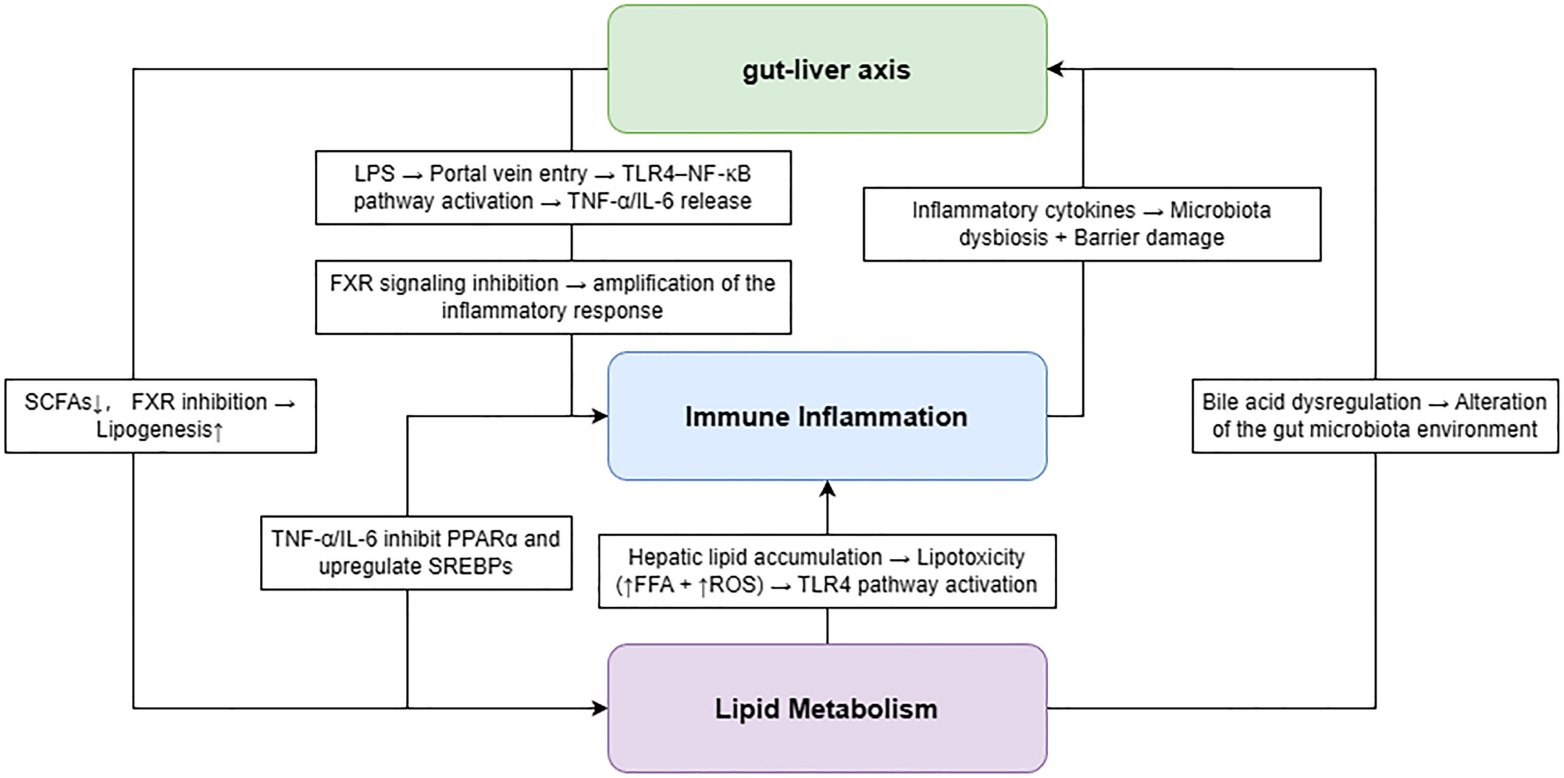
Schematic diagram of the interactions among lipid metabolism, inflammation, and the gut-liver axis.

Mitochondria play a central role in hepatic energy metabolism and fatty acid oxidation. In MASLD, excessive lipid accumulation and metabolic overload impair mitochondrial function, leading to reduced β-oxidation efficiency and increased production of reactive oxygen species (ROS) (60). The accumulation of ROS induces oxidative stress, which damages cellular structures, including lipids, proteins, and DNA, thereby promoting hepatocyte injury and disease progression (**Figure 4**).

Oxidative stress further amplifies inflammatory responses by activating stress-related signaling pathways, including NF-κB and JNK, thereby linking metabolic disturbances to immune-inflammatory activation (61). In parallel, lipid overload and metabolic stress can trigger ER stress through the accumulation of misfolded proteins. The activation of the unfolded protein response (UPR) initially serves as a protective mechanism; however, persistent ER stress leads to hepatocyte apoptosis and exacerbates inflammation and fibrosis (62).

Importantly, these mechanisms do not operate in isolation but interact dynamically with previously described pathways. For example, mitochondrial dysfunction exacerbates lipid metabolism disorders, while oxidative stress and ER stress further amplify immune-inflammatory responses and gut–liver axis dysregulation (63). Together, these interconnected processes drive the progression of MASLD from simple steatosis to MASH and advanced fibrosis.

## 4 Conclusion

This review systematically synthesizes current evidence on the key risk factors and pathogenic mechanisms of MASLD. Importantly, beyond a descriptive summary, this work provides an integrated framework that links major metabolic risk factors—including obesity, non-obese phenotypes, type 2 diabetes, and genetic susceptibility—with three central pathogenic pathways: hepatic lipid metabolism imbalance, immune-inflammatory activation, and disruption of the gut–liver axis. In addition, this review highlights emerging and underexplored aspects of MASLD, particularly the growing recognition of lean MASLD and the influence of population-specific genetic susceptibility, with a special focus on Asian populations. By integrating these perspectives, this study offers a more comprehensive understanding of the heterogeneity and complexity of MASLD compared with conventional reviews. Despite these advances, certain limitations remain in current mechanistic research. Future studies should further explore the interactions among metabolic factors, genetic background, and environmental influences, as well as identify potential therapeutic targets within key pathways such as the gut–liver axis and immune-inflammatory networks. Overall, this review provides a more structured and clinically relevant synthesis to support precision diagnosis, stratified treatment, and improved prevention strategies for MASLD.
